# Cost-Effectiveness Analysis of a Community Health Worker Intervention for Low-Income Hispanic Adults with Diabetes

**DOI:** 10.5888/pcd9.120074

**Published:** 2012-08-23

**Authors:** H. Shelton Brown, Kimberly J. Wilson, José A. Pagán, Christine M. Arcari, Martha Martinez, Kirk Smith, Belinda Reininger

**Affiliations:** Author Affiliations: H. Shelton Brown, III, University of Texas Health Science Center School of Public Health, Austin, Texas; José A. Pagán, University of North Texas Health Science Center, Fort Worth, Texas; Christine M. Arcari, Kirk Smith, University of Texas Medical Branch, Galveston, Texas; Martha Martinez, University of Texas Health Science Center, San Antonio, Texas; Belinda Reininger, University of Texas Health Science Center School of Public Health, Brownsville, Texas.

## Abstract

**Introduction:**

The objective of our study was to estimate the long-term cost-effectiveness of a lifestyle modification program led by community health workers (CHWs) for low-income Hispanic adults with type 2 diabetes.

**Methods:**

We forecasted disease outcomes, quality-adjusted life years (QALYs) gained, and lifetime costs associated with attaining different hemoglobin A1c (A1c) levels. Outcomes were projected 20 years into the future and discounted at a 3.0% rate. Sensitivity analyses were conducted to assess the extent to which our results were dependent on assumptions related to program effectiveness, projected years, discount rates, and costs.

**Results:**

The incremental cost-effectiveness ratio of the intervention ranged from $10,995 to $33,319 per QALY gained when compared with usual care. The intervention was particularly cost-effective for adults with high glycemic levels (A1c > 9%). The results are robust to changes in multiple parameters.

**Conclusion:**

The CHW program was cost-effective. This study adds to the evidence that culturally sensitive lifestyle modification programs to control diabetes can be a cost-effective way to improve health among Hispanics with diabetes, particularly among those with high A1c levels.

## Introduction

Diabetes accounts for an estimated 11% of health care costs in the United States; 8.3% of the US population has diabetes (1). Adults over the age of 50 with diabetes are more likely to develop cardiovascular disease and have decreased life expectancy of 7.5 to 8.2 years compared with those without diabetes (2). Although the Hispanic paradox – that Hispanics have greater life expectancy than their non-Hispanic counterparts – is well-documented (3), Hispanics are disproportionately affected by diabetes (4,5). The prevalence of diabetes in US adult Hispanics is 13.3% compared with 7.1% for non-Hispanic whites (6). Hispanics with diabetes have hemoglobin A1c (A1c) levels that are 0.5 percentage points higher than non-Hispanic whites (7). In one study, Hispanic males had mean A1c levels 9.3% higher than non-Hispanic white males (8.2% vs 7.5%), and Hispanic females had mean A1c levels that were 3.9% higher than non-Hispanic white females (7.9% vs. 7.6%) (8). Hispanics are 1.5 times more likely to die from diabetes than their non-Hispanic white counterparts (9). Reasons cited for these disparities include less access to diabetes-specific care, language barriers, beliefs about diabetes, and health insurance coverage status (7).

A 1% reduction in A1c levels has been correlated with a 21% reduction in vascular complications in people with diabetes, resulting in fewer complications and reduced lifetime health care costs (10). Additionally, researchers have found that among the 2.1 million people in the United States with type 2 diabetes, those with good glycemic control (A1c 7 or less) had direct diabetes-related medical costs that were 16% lower than those with fair glycemic control (A1c ≥7 - ≤9) and 20% lower than those with poor glycemic control (A1c > 9) (11).

Community health workers (CHWs) are effective in motivating sustained lifestyle changes in Hispanics with diabetes. CHW interventions improve knowledge about this chronic health condition (12,13), self-management (13–15), and self-efficacy (13). CHW interventions have also resulted in significant reductions in A1c levels (12,13,15,16). Although research has analyzed the cost-effectiveness of CHW-based diabetes management interventions delivered in primary care settings (17,18), we know of no cost-effectiveness studies of diabetes self-care interventions that involve CHWs delivering home-based counseling and education. Given this population’s lack of access to traditional health care services, the provision of home-based diabetes self-management education is likely to add substantial value to, and improve the effectiveness of, diabetes management initiatives. However, program delivery in patient homes necessitates added costs. This study analyzes the cost-effectiveness of a diabetes management intervention for Hispanics delivered in both primary care and home settings. The objective of our study was to estimate the long-term cost-effectiveness of a lifestyle modification program led by community CHWs for low-income Hispanic adults with type 2 diabetes.

## Methods

### Priority population

The priority population for this study was Hispanic adults aged 18 years or older with type 2 diabetes who were patients at the Mercy Clinic in Laredo, Texas, from October 2009 to January 2010. Laredo is in south Texas on the Mexico border, in Webb County. About 96% of the population of Webb County is of Hispanic origin, and median household income is $37,140 (19). Almost half of adults aged 18 to 64 in the Laredo metropolitan statistical area (MSA) lack health insurance coverage (20).

Analysis inclusion prerequisites were a diagnosis of type 2 diabetes, availability of baseline and follow-up A1c readings, and evidence of program participation. Forty-six participants met these criteria. Of the 46, 16 had baseline A1c levels at or below 7%, indicating good glycemic control, and were therefore excluded from the study group. The sample analyzed consists of the remaining 30 participants.

### Intervention components

The University of Texas Community Outreach (UTCO) intervention is an ongoing community-based diabetes education and self-management program that uses community partnerships and trained CHWs to reach participants. Four Texas counties, including Webb County, host UTCO programs. We used first-wave evaluation data (follow-up data collected through January 15, 2011) from the Webb County UTCO program for this analysis.

Mercy Clinic, the primary Webb County partner in the UTCO program, provides primary health care, health education, and social services to the financially disadvantaged and medically underserved population. Intervention components include home-based CHW visits, classroom health education classes, nutrition classes, exercise classes, and counseling sessions. The nutrition classes teach new diabetes management skills. Exercise classes provide venues and social support for physical activity. Counseling sessions provide targeted guidance on individual issues participants have in managing their diabetes. Home visits provide reinforcement of themes covered in classes and support in creating cues to action. CHWs bring schedules of upcoming classes to home visits, provide guidance on which sessions participants should give priority to, and assist participants in overcoming barriers related to effective diabetes management.

Five state-certified CHWs and a nurse educator are the primary UTCO service providers. Four of the 5 CHWs were previously employed by Mercy Clinic, and external funding of the UTCO program covered the project director’s salary and enabled hiring of a fifth CHW and a nurse practitioner. CHWs received training through the UT Health Science Center School of Public Health, Brownsville Regional Campus, on leading nutrition and exercise classes and on data-gathering protocols. All CHWs, who are called *promotoras* in the Hispanic community, are certified through the Texas Department of Health and Human Services’ CHW training program. The CHWs’ primary role is to develop relationships with participants to provide individualized guidance, one-on-one diabetes education, and counseling aimed at increasing participants’ ability and self-efficacy to manage their diabetes. Additionally, CHWs and the nurse educator teach participants in group environments how to manage their diabetes, improve their nutrition, and lose weight. Several volunteers, including a dietitian, a certified Zumba instructor, and students from Texas A&M International University (TAMIU), assist with teaching nutrition classes, leading exercise classes, and facilitating group counseling sessions.

To be included in the analysis, participants had to have received at least 1 home visit in addition to having a baseline A1c reading above 7.0. We set these criteria for several reasons 1): many people attended classes only, and our primary focus was the combination of CHWs in clinical (classes) and community (home) settings; 2) home visits are the most expensive component of the intervention, and we wanted to include these costs in our analysis; 3) we hypothesized that these in-home activities would be key components to the program’s success and to sustained outcomes; and 4) the agreement between Mercy Clinic and participants included attendance at both classes and home visits. All participants attended nutrition and health education classes (mean number of classes attended, 8.3); 77% attended exercise classes (mean number of classes attended, 4.2); and 33% attended counseling sessions (mean number of sessions attended, 4.3). Follow-up A1c readings were taken periodically, ranging from 37 to 565 days (mean, 75 days) for the sample.

### Costs

We assessed the cost-effectiveness of the UTCO intervention in this location from a societal perspective. The societal perspective includes all measurable opportunity costs, representing all groups affected by a program. To assess program costs, we included staff time and valuation of volunteer and participant time for home visits, educational classes, counseling sessions, exercise classes, class-related materials, and mileage related to home visits.

Mercy Clinic paid staff salaries and for program supplies. We used the Bureau of Labor Statistics Metropolitan and Non-Metropolitan Area Occupational Employment and Wage Estimates for the Laredo MSA (21) to value volunteer and participant time. Where volunteer occupation was known (eg, dietitian), we used the mean wage for that occupation, and we included the value of fringe benefits and taxes for clinic staff and volunteers. We used the Texas minimum wage ($7.25 hourly) to value student volunteer time and the average hourly wage rate for the Laredo MSA ($16.14) to value the time of study participants. This average rate was likely higher than what study participants were earning in their jobs. We added only Federal Insurance Contributions Act (FICA) taxes to participant base wages. To calculate the average cost per home visit, we used information gathered from CHW interviews about the typical length of a home visit, the time needed to schedule visits, and travel time to and from appointments. Estimated average mileage expense was assessed at $0.51 per mile. Initial home visits took, on average, twice as long as subsequent visits but involved the same amount of scheduling and travel time; we factored this into total home visit expenses and assigned a value of $80.59 for initial home visits and $48.16 for follow-up visits.

We calculated a composite fixed cost per class from information gathered from Mercy Clinic records on time and resources needed to deliver each type of class. We weighted the cost for each type of education class by the frequency with which the class was offered and summed them to derive an average class cost. We similarly developed composite per-session fixed costs for exercise classes and counseling sessions. Total costs for each activity category were the sum of the fixed costs of the classes and aggregate attendee time costs. This composite fixed cost was multiplied by the total number of classes or sessions; to this product we added the average attendance and the per-participant time cost of attendance to derive the time costs of attendees. Ten percent of the total CHW training expense was included in the intervention costs. The time costs for staff, volunteers, and participants; the class and session time; mileage costs; resource costs; and the CHW training expenses were summed to derive a total program cost for the sample, resulting in a per-participant program cost of $1,175.63 for the 18-month period. These cost calculations ignore economies of scale that would accrue from the program being run at a capacity more reflective of real-world situations. To create a real-world cost structure, we used the original fixed and variable costs for each activity, but increased average attendance levels to reflect data collected on actual class attendance (ie, which included participants not used in the sample); we then calculated intervention costs based on the prevalence of diabetes in the Archimedes Model (Archimedes, Inc, San Francisco, California) (n = 6,551) (Table 1).

**Table 1:** Trial Versus Real World Scenarios, Cost-Effectiveness Analysis of a Community Health Worker Intervention for Hispanic Adults with Diabetes, October 2009–January 2010

Category	Trial Scenario, n = 30^a^		Real-World Scenario, n = 6,551^b^		Real-World Scenario, Ongoing, n = 6,551^c^
	Average Attendance	Program Costs, $	Average Attendance	Program Costs, $	Ongoing Costs, $
Educational classes	1.7	15,995	6	1,883,361	941,681
Exercise classes	1.6	4,524	7	513,396	256,698
Counseling sessions	1.9	2,247	3	366,955	183,477
Home visits^d^	7.8^c^	12,242	7.8^a^	2,673,319	NA
CHW training	NA	261	NA	57,155	NA
Total costs	NA	35,269	NA	5,494,185	1,381,856

Abbreviation: CHW, community health worker; NA, not applicable.

^a ^Calculates costs based on the original sample size of 30, and the per-class attendance by participants in the sample.

^b ^Reflects attendance levels and associated costs that are likely if the program served a larger number of people. The number used here is the number of people in the Archimedes data set who reflect the program’s target demographics.

^c ^The annual costs beyond the formal 18-month program borne by participants as a result of lifestyle changes made during the program.

^d ^Average number of home visits that participants received.

Finally, because diabetes management is a long-term process, we estimated ongoing postintervention costs assuming continued participation in educational classes, counseling, and exercise activities at 50% of intervention levels over the 3 periods of our projections (5, 10, and 20 years). This resulted in annual post-intervention costs of $140.63 per participant. We used data from the 2006 Medicare Current Beneficiary Survey for medical costs (22); lifestyle change costs were taken from a systematic review of costs of major US commercial weight loss programs (23).

### Projecting outcomes and medical costs

We used the Archimedes model to project the intervention’s incremental lifetime health outcomes and related expenditures based on changes in A1c levels. It simulates the effects of interventions by generating multiyear forecasts of predicted disease outcomes, health behaviors, and health care costs relative to a hypothetical control group with similar demographic characteristics. Studies have independently validated the model for diabetes outcomes research (24).

We applied data from the Archimedes Cardio-Metabolic Risk (CMR) dataset, which includes data from a simulated US representative sample of 100,000 people aged 30 to 85 years (25) to our study sample of 6,551. The CMR dataset includes the results of 19 simulated controlled clinical trials comparing standard care (defined as current levels of care in the US population) with a set of health management interventions targeting cardio-metabolic risk, including glycemic control (25).

For the primary analysis, we grouped study participants into 2 cohorts: those whose diabetes was brought under control (ie, follow-up A1c reading was ≤7%) and those who were still out of control at follow-up (A1c >7%) (Table 2). During the intervention, the diabetes of 60% of the subjects was brought under control. We assumed that the sample’s levels of glycemic control would fluctuate over time. When A1c becomes uncontrolled, the intervention in the CMR dataset is designed to bring A1c under control. On the other hand, A1c in the dataset’s control group is uncontrolled at baseline and can vary over time; however, there is no specific intervention apart from usual care to reduce A1c to a target level. We discounted program costs, ongoing participation costs, medical costs, and quality-adjusted life years (QALYs) at a 3% rate. Costs are given in 2010 dollars. Archimedes uses disutility weights for diabetes-related disorders and illnesses from 2 studies. One (26) used responses from the 2000-2002 Medical Expenditure Panel Survey (27) and the EQ-5D questionnaire (28) from a nationally representative sample of 38,678 adults to develop health-related quality of life scores for 95 chronic diseases with ICD-9 codes. The other, by Coffey and colleagues (29), used the Self-Administered Quality of Well-Being index to calculate health utility scores for 2,048 people with type 1 and type 2 diabetes. A life-year with none of the 95 chronic diseases present receives a health utility score of 1; the presence of 1 or more of the chronic diseases reduces the score in accordance with its additive contribution to health disutility.

**Table 2:** Cohort Characteristics and Cost-Effectiveness Analysis, Community Health Worker Intervention for Hispanic Adults with Diabetes, October 2009–January 2010

Characteristic		Glycemic Levels Controlled, mean (SD),^a^ n = 18	Glycemic Levels Not Controlled, Mean (SD)^a^, n = 12
Sex	Male	16	10
	Female	2	2
Age		52.06 (11.11)	48.82 (10.99)
Baseline A1c		9.56 (2.53)	10.5 (2.39)
Ending A1c		6.29 (0.40)	8.55 (1.33)
Home visits		8.61 (5.57)	6.58 (6.08)
Attended classroom education		7.56 (3.48)	9.33 (9.04)
Attended exercise classes		3.33 (3.12)	3.08 (3.06)
Attended counseling sessions		1.5 (2.33)	1.33 (2.35)

Abbreviation: SD, standard deviation; A1c, hemoglobin A1c.

^a^ Presented as frequencies.

We applied the discount rate to the program and ongoing costs to derive the net present value of intervention costs. We used the Archimedes simulation model to calculate the long-term differential medical costs and QALYs of people with type 2 diabetes who do not have access to the program, who we used as controls. We summed the long-term medical costs and net present-value intervention costs, subtracted the standard-care medical costs, and divided by the difference in QALYs between each intervention and the simulated control group to derive incremental cost effectiveness ratios (ICERs).

## Results

The effects of the UTCO intervention are reflected in clinical outcomes for a cohort of participants whose A1c levels at the beginning of the intervention were above 7%, but fell to 7% or below during the course of the 18-month intervention (Table 3). We used the real-world cost structure and assumed ongoing annual costs at the 50% level. On the basis of our estimates using the Archimedes model, the UTCO intervention is expected to reduce the risk of a myocardial infarction by 2.6%, foot ulcers by 5.6%, and foot amputation by 3.5%. A1c levels will fall by 11.7%, and 413.52 life years will be gained through the intervention over a 20-year period. After accounting for health disutility weights, the UTCO intervention results in an incremental gain of 394.92 QALYs (Table 4).

**Table 3:** Expected Outcomes by Age Group for Participants Receiving Intervention, Community Health Worker Intervention for Hispanic Adults with Diabetes, October 2009–January 2010

Disorder	Standard Care, %				Absolute Difference over 20 y,^a^ %			
	30-49 y	50-64 y	65-84 y	All Ages	30-50 y	50-65 y	65-84 y	All Ages
Bilateral blindness	3.30	3.56	4.73	3.87	–0.32	0.05	–0.03	–0.08
CHD death	2.94	3.64	5.39	4.02	–0.40	–0.40	–0.82	–0.54
Death	19.30	42.53	81.50	48.84	–0.49	–0.72	–0.37	–0.55
ESRD	0.30	1.84	18.04	6.70	0.00	0.05	0.92	0.32
Foot amputation	17.39	18.00	18.09	17.86	–3.81	–3.69	–2.92	–3.47
Foot ulcer	46.94	45.37	33.43	41.92	–6.68	–6.22	–3.95	–5.60
FPG	11.65	7.57	9.24	3.10	–2.28	–1.38	–1.62	–0.58
MI	13.43	22.81	23.63	20.48	–1.69	–2.75	–3.20	–2.60
PDR	46.46	44.79	41.16	44.06	–3.69	–1.95	–0.60	–1.99

Abbreviations: CHD, coronary heart disease; ESRD, end stage renal disease; FPG, fasting plasma glucose; MI, myocardial infarction; PDR, proliferative diabetic retinopathy.

^a^ Absolute difference between standard care and care received through intervention over a 20-year period.

**Table 4:** Incremental Cost-Effectiveness, Community Health Worker Intervention for Hispanic Adults with Diabetes, October 2009–January 2010

Age Range	30-49 y	50-64 y	65-84 y	All Ages
Life years	69.96	150.54	193.20	413.52
Undiscounted QALYs	133.70	225.84	204.23	563.64
QALYs, discounted 3%	90.92	157.15	146.92	394.92
Cost per QALY – 20 y, $	39,021	30,786	33,103	33,319
Cost per QALY – 10 y, $	NA	NA	NA	56,009
Cost per QALY – 5 y, $	NA	NA	NA	130,272

Abbreviations: QALY, quality-adjusted life year; NA, not applicable.

The resultant ICER for a 20-year period was $33,319 per QALY gained for the entire population relative to standard care (Table 4). The intervention was most cost-effective for those aged 50 to 65 years, with a ratio of $30,786 per QALY gained. The intervention had an ICER of $130,272 per QALY gained over a 5-year period and $56,009 per QALY gained over a 10-year period.

We also conducted sensitivity analyses to assess how our results would differ under different assumptions. The results stand up to variation for all parameters except when lowering program effectiveness from 60% to 41%, which increased the ICER per QALY gained to $51,462. Raising the effectiveness to 73% lowered the ICER to $28,093 per QALY gained. Adjusting the discount rate to 0% and to 6% resulted in ICERs per QALY gained of $30,026 and $37,473, respectively. Finally, lowering annual costs from 50% of program costs to 25% resulted in an ICER of $21,977 per QALY gained; increasing them to 75% resulted in $45,696 per QALY gained.

Eighty percent of the cohort that entered the program with an A1c level above 9% lowered their A1c levels to below 9% at follow-up. For this cohort, the ICER was $10,995 per QALY gained. This is largely a function of the long-term health care cost savings accrued. Decreasing effectiveness to 60% raised the ICER to $18,680 per QALY gained; raising effectiveness to 100% lowered it to $6,384 per QALY gained. Setting the discount rate for costs as well as QALYs gained to 0% lowered the ICER to $9,980 per QALY gained, and setting it at 6% raised the ICER to $12,405 per QALY gained. Finally, lowering continuance costs to 25% lowered the ICER to $2,156 per QALY gained; raising these costs to 75% resulted in an ICER of $19,834 per QALY gained.

## Discussion

The 20-year cost-effectiveness of the UTCO CHW-based diabetes management program was $33,319 per QALY gained for the cohort of low-income Hispanics in our primary analysis. The ratios were $56,009 and $130,272 for the 10- and 5-year projections, respectively. Because diabetes is a disease whose full effects occur over the long term, the higher ICERs based on shorter term projections are not surprising.

From a health care standpoint, interventions to prevent or treat diabetes are deemed cost effective if they fall under the threshold of $50,000 per QALY gained (30). Thus, the UTCO intervention’s cost per additional QALY over 20 years compares favorably to other interventions, despite its societal perspective.

Our estimates also compare favorably to cost-effectiveness studies of other lifestyle modification interventions for people with diabetes. Eddy and colleagues modeled the cost-effectiveness of the Diabetes Prevention Program (DPP) lifestyle intervention and found an ICER of $24,500 per QALY gained when compared with standard care over a 30-year period (31). Herman and colleagues used a Markov model to estimate the cost effectiveness of the DPP lifestyle modification intervention and reported an ICER of $8,800 per QALY gained (32). A Netherlands-based study of a 3-year, 1-time intensive lifestyle intervention targeting obese adults with impaired glucose tolerance found the intervention to be cost effective in reducing new cases of diabetes over a 20-year period (33). Gilmer and colleagues studied the impact of diabetes case management in a similar population and found the intervention to be cost effective for the uninsured and publicly insured populations (17).

Finally, our results for the cohort with baseline A1c above 7% likely underestimate the health gains accruing from the intervention for 2 reasons. First, the average A1c level at baseline was higher (9.55%) for our predominantly Hispanic sample than that of the US representative population in the Archimedes CMR forecast dataset (8.64%). Second, our analysis of the cohort with baseline A1c levels above 9% demonstrates greater cost effectiveness than the above-7% cohort, despite projecting reductions in A1c to only 9% or lower, 2 percentage points above the standard for diabetes control. It appears that it is more cost effective to focus resources on reducing the glycemic levels of people whose A1c levels are out of control to 9% as compared with allocating resources to reduce A1c levels to 7% for the population with A1c levels between 7% and 9%.

The cost effectiveness of the UTCO intervention appears to be higher, or at least comparable, to other lifestyle and health-management interventions targeting Hispanics. CHWs are instrumental in gaining compliance with the program through relationships, likely because of the rapport they develop with clients. What seems to make CHWs so effective is that they provide services in the communities in which they live while also being peers of program participants. In the case of diabetes interventions, many CHWs have diabetes themselves, resulting in greater rapport and trust being established with program participants. They also provide more personalized interaction than traditional health care professionals, resulting in improved access to health care and better self-efficacy (33). Our study suggests that CHWs are not only effective at improving health and health care quality outcomes, but seem to be able to do so, from a societal perspective, in a cost-effective way.
